# Effective treatment of alkaline Cr(VI) contaminated leachate using a novel Pd-bionanocatalyst: Impact of electron donor and aqueous geochemistry

**DOI:** 10.1016/j.apcatb.2015.01.017

**Published:** 2015-07

**Authors:** Mathew P. Watts, Victoria S. Coker, Stephen A. Parry, Russell A.P. Thomas, Robert Kalin, Jonathan R. Lloyd

**Affiliations:** a School of Earth, Atmospheric and Environmental Sciences, Williamson Research Centre for Molecular Environmental Science, The University of Manchester, Manchester M13 9PL, UK; b Diamond Light Source, Chilton, Didcot, Oxfordshire OX11 ODE, UK; c Parsons Brinckerhoff, Queen Victoria House, Redland Hill, Bristol, UK; d Department of Civil and Environmental Engineering, James Weir Building, University of Strathclyde, Glasgow G1 1XJ, UK

**Keywords:** Cr(VI), Magnetite, Remediation, Alkaline, Inactivation

## Abstract

Palladium catalysts offer the potential for the effective treatment of a variety of priority reducible pollutants in natural waters. In this study, microbially synthesized magnetite nanoparticles were functionalized with Pd(0), creating a highly reactive, magnetically recoverable, nano-scale catalyst (Pd-BnM). This was then investigated for the treatment of model Cr(VI) contaminated solutions at a range of pH values, and also alkaline Cr(VI) contaminated leachates from chromite ore processing residue (COPR); a contaminant issue of global concern. The sample of COPR used in this study was obtained from a site in Glasgow, UK, where extensive Cr(VI) contamination has been reported. In initial experiments Pd-BnM was supplied with H_2_ gas or formate as electron donors, and Cr(VI) removal from model synthetic solutions was quantified at various pH values (2–12). Effective removal was noted at neutral to environmentally relevant alkaline (pH 12) pH values, while the use of formate as an electron donor resulted in loss of performance under acidic conditions (pH 2). Reaction kinetics were then assessed with increasing Pd-BnM loading in both model pH 12 Cr(VI) solutions and the COPR leachate. When formate was used as the electron donor for Pd-BnM, to treat COPR leachate, there was significant inhibition of Cr(VI) removal. In contrast, a promotion of reaction rate, was observed when H_2_ was employed. Upon sustained reaction with model Cr(VI) solutions, in the presence of excess electron donor (formate or H_2_), appreciable quantities of Cr(VI) were removed before eventual inactivation of the catalyst. Faster onset of inactivation was reported in the COPR leachates, removing 4% and 64% of Cr(VI) observed from model Cr(VI) solutions, when formate and H_2_ were used as electron donors, respectively. XAS, TEM-EDX and XPS analysis of the catalysts that had been inactivated in the model solution, showed that the surface had an extensive covering of reduced Cr(III), most likely as a CrOOH phase. COPR reacted catalysts recorded a lower abundance of Cr(III) alongside a high abundance of the leachate components Ca and Si, implicating these elements in the faster onset of inactivation.

## Highlights

• Pd(0) supported on biogenic nano-magnetite highly reactive for reduction of aqueous Cr(VI).
• Efficiency of aqueous Cr(VI) removal dependent upon geochemical matrix and electron donor used.
• Catalyst inactivation occurs by Cr(III)OOH precipitation in chemically simple solutions.
• In complex Cr(VI) contaminated waters precipitation of Ca and Si phases increases inactivation.
• Cr(VI) removal from contaminated waters far higher than conventional nanoparticle treatments.

## Graphical abstract

**Figure:**
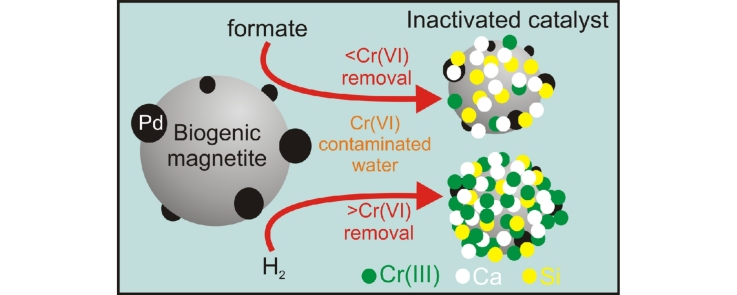

## 1 Introduction

An emerging technology for the treatment of a variety of reducible pollutants is the utilization of palladium metal (Pd(0)) catalysts [1]. Heterogeneous Pd(0) catalysts are able to dissociatively absorb reactive hydrogen, which can drive hydrogenation reactions with adsorbed target compounds [2–4]. Although molecular hydrogen (H_2_) has been the most extensively used electron donor during Pd(0)-mediated catalysis, it is poorly soluble in water and other more soluble forms of electron donor, typically simple organic acids such as formate (HCOO^−^), have been employed as an alternative [5]. This catalytic approach to contaminant remediation has been demonstrated to be effective towards a variety of key contaminants, including chlorinated hydrocarbons [6–10], nitrobenzene [11], nitrate [12,13] and Cr(VI) [14–19].

A wide variety of Pd(0) catalysts have been developed, typically supported upon a carrier particle or in combination with a promoter metal to improve recoverability and increase reactivity [20,21]. Microbial synthesis techniques have also been employed, through the direct enzymatic reduction of a Pd(II) solution by bacterial cells, to form biomass supported Pd(0) [22–26]. Further to this, a novel whole-cell mediated method was developed; using a model Fe(III)-reducing bacterium to reduce an Fe(III) oxyhydroxide, producing nano-scale magnetite with a narrow size distribution with controllable reactivity and particle size [27]. The biogenic nano-magnetite was then used to abiotically reduce aqueous Pd(II) to create magnetically recoverable magnetite supported Pd(0) nanoparticles [28]. This novel nano-scale heterostructure was used initially to catalyze organic coupling reactions [28], and subsequently to treat Cr(VI) in neutral pH test solutions [17].

A sizable Cr(VI) contamination problem has resulted from the poorly regulated disposal of chromite ore processing residue (COPR), as a waste product of the “high lime” chromite ore processing technique [29,30]. Upon saturation with water, COPR yields a highly alkaline (pH 10–12.5) leachate which, due to the relatively high solubility of most Cr(VI) minerals, can yield high concentrations of aqueous Cr(VI) [31,32]. Specifically in Glasgow, UK, >2 million tons of COPR was disposed of, leading to extensive contamination of ground and surface waters with Cr(VI) at concentrations up to 100 mg L^−1^ [33,34]. Cr(VI) typically forms soluble oxyanions [35,36], which are regarded as toxic and potential carcinogens [37]. As a result, an upper limit of 0.05 mg L^−1^ Cr(VI) in drinking water has been set by the World Health Organization [38]. The reduced Cr(III) state, in contrast, is regarded as non-toxic and far less soluble, forming a range of stable oxides and (oxy) hydroxides [39]. The reductive stabilization of the toxic Cr(VI) to non-toxic Cr(III), is therefore the aim of most remediation strategies [40]. However, remediation of COPR related Cr(VI) has proven problematic, due to the large quantities of materials involved, and the adverse alkaline pH that often impacts on the efficiency of conventional chemical treatments [32,41,42]. A recent study employing biogenic nano-scale magnetite and nano-scale zero valent iron highlighted the potential for nano-particle treatment of COPR and its groundwater [43]. Significantly the electron donating capacity of these particles was limited by the supply of reactive Fe, and passivation of the reactive surface by the reduced Cr(III) and groundwater chemical components.

Using Pd(0) functionalized nano-scale biomagnetite (Pd-BnM) [28], this study aims to extend our understanding of catalytic Cr(VI) reduction to the environmentally relevant alkaline pH range, and to assess its applicability to the treatment of COPR leachates. As formate has been previously proposed as an alternative electron donor to H_2_ gas for pollutant reduction [5,16], the performance of both electron donors was assessed in experiments using model alkaline Cr(VI) solutions and a COPR leachate. Inactivation of the catalyst is also of great concern when considering catalyst applications, therefore this was investigated after reaction with the model and COPR solutions, using a variety of spectroscopic and nano-imaging techniques.

## 2 Experimental

### 2.1 Biogenic magnetite synthesis and functionalization with Pd(0)

Biogenic magnetite was synthesized by the dissimilatory reduction of ferrihydrite by a culture of *Geobacter sulfurreducens* according to the method of [44], detailed in the Supporting Information (SI Text S1.). The surface of the bio-magnetite was functionalized with 5 mol % Pd of magnetite (∼2% by mass Pd of Pd-BnM) via surface Fe(II)-mediated reductive precipitation from a Na_2_Pd(II)Cl_4_ solution, according with the method of [28]. The solution was agitated, under an N_2_ atmosphere, for a 12 h period, after which excess ions were removed by washing with 18.2 MΩ water and the resulting slurry was stored under an N_2_ atmosphere until use.

### 2.2 Cr(VI) solutions

A model Cr(VI) solution was prepared using K_2_CrO_4_ dissolved in 18.2 MΩ water, and the pH adjusted as required using HCl and NaOH. A sample of COPR was obtained from a borehole in the south east of Glasgow and stored in a sterile container in the dark at 10 °C until use. To obtain a leachate from the COPR, 100 g of field wet solid was suspended in 1 L of ultrapure water for 24 h at 20 °C in the dark. The homogenized slurry was passed through a 0.22 μm filter and analyzed for its major aqueous chemical components. Analysis of the COPR leachate showed it to be highly alkaline (pH 11.98) and dominated by Ca (15 mM) and CO_3_^2−^ (13 mM), with a minor component of Si (0.06 mM). There was a significant concentration of Cr (0.5 mM), entirely in the Cr(VI) oxidation state (S.I. Table S1).

### 2.3 Batch Cr(VI) removal experiments

All batch experiments were carried out using acid washed 120 mL serum bottles containing 100 mL solution, sealed using butyl rubber stoppers and aluminum crimps. The bottles were flushed using pressurized N_2_ gas passed through a 0.22 μm cut-off filter. The electron donor was supplied in the form of a Na-formate solution spike or by sparging with pressurized H_2_ gas passed until saturation, leaving 20 mL pure H_2_ as headspace. Pd-BnM additions were made to the bottles by the injection of a known concentration of a stock Pd-BnM suspension, using a syringe flushed with N_2_. Samples were removed from the serum bottles using an N_2_-degassed syringe and centrifuged (Sigma 1–14 Microfuge) at 13,000 g for 4 min, and a sub sample of the supernatant taken for aqueous analysis. Throughout the experiments, serum bottles were maintained in the dark at 20 °C on a rolling shaker.

To better characterize the capacity of Pd-BnM for Cr(VI) removal, initial experiments sought to assess the effect of pH and varying formate concentrations on the reaction. The pH experiment was conducted with a model Cr(VI) solution (1 mM) and a constant Pd-BnM addition of 0.32 g L^−1^, employing saturation with H_2_ or 100 mM formate as electron donors, with the starting pH adjusted to a range of values between 2–12. The variable formate concentration experiment used a model solution of 0.5 mM Cr(VI) at pH 12 and increasing formate concentrations (0–200 mM), and 0.24 g L^−1^ Pd-BnM.

To assess Cr(VI) removal kinetics, a series of batch experiments were conducted, containing varying Pd-BnM loading, using a pH 12 Cr(VI) (0.5 mM) solution and the COPR leachate. The electron donors for these batch experiments were either 100 mM formate or H_2_ gas.

The maximum removal of Cr(VI) reached, prior to deactivation of the Pd-BnM catalyst, was measured in batch systems with excess Cr(VI), as model solutions or COPR leachate, and electron donor; 1 M formate or periodic re-saturation with H_2_ gas. These experiments were maintained for 2 weeks and sampled periodically, to ensure Cr(VI) removal had ceased, prior to sampling of the catalyst for solid phase analysis.

### 2.4 Aqueous phase analyses

Anion concentrations were determined by ion chromatography (IC). Analysis of formate (sample injection volume of 0.4 μL) was performed using a Dinoex ICS5000 Dual Channel I.C. fitted with a Dionex Capillary AS11-HC 4 u (250 × 0.4 mm) column. A 1 mM to 36 mM KOH gradient mobile phase was applied over 40 minutes at a flow rate of 0.015 mL min^−1^ and a back pressure of 3400 psi. A Dinoex AS18 microbore (250 × 2 mm) column was used for analysis of CO_3_^2−^ (sample injection volume of 10 μL), using a mobile phase of 30 mM KOH at a flow rate of 0.25 mL min^−1^ and a back pressure of 14 MPa.

Analysis of the concentration of Si, Cr and Ca, in acidified aqueous samples (2% HNO_3_), were performed using inductively coupled plasma atomic emission spectroscopy (ICP-AES) on a PerkinElmer Optima 5300 dual view ICP-AES.

Aqueous Cr(VI) concentration was determined by a spectrophotometric method using 1,5-diphenylcarbazide (DPC) [45]. Analysis was performed on a Jenway 6715 UV–vis spectrophotometer, using daily calibration curves of known Cr(VI) concentration standards.

The pH values of aqueous samples was determined using a Denver Instrument UB-10 meter and a P Cole Parmer 5990–45CCP probe, calibrated to relevant buffers.

### 2.5 Solid phase analyses

Transmission electron microscopy (TEM) imaging and elemental mapping of the un-reacted and model solution reacted samples were performed on a FEI Tecnai TF20 microscope at a beam voltage of 200 KeV, equipped with an Oxford Instruments INCA 350/80 mm X-Max SDD detector for energy dispersive X-ray analysis (EDX), field emission gun (FEG), high angle annular dark field (HAADF) detector, and a GatanOrius SC600A CCD camera. COPR leachate-reacted samples were analyzed on a Philips CM200 FEG TEM, equipped with an Oxford Instruments X-Max 80 mm^2^ SDD INCA EDX. Prior to analysis, the dried sample was re-suspended in ethanol and droplets placed upon an Agar Scientific Holey Carbon Film grid and allowed to dry.

X-ray photoelectron spectroscopy (XPS) data of the un-reacted Pd-BnM were collected on a VG Escalab 250 instrument, using a monochromatic Al Kα X-ray source, with an analyzer pass energy of 20 eV and a total energy resolution of ∼0.9 eV, using a flood gun to create uniform charge neutralization. The Cr(VI) reacted Pd-BnM samples were analyzed on a Kratos Axis Ultra spectrometer with a monochromated Al Kα X-ray source, with an analyzer pass energy of 80 eV (wide scans) and 20 eV (narrow scans), with a total energy resolution of 1.2 and 0.6 eV, respectively. Both systems had a base pressure of 5 × 10^−10^ mbar. Prior to analysis, all samples were dried and manipulated in an N_2_ glove box and loaded in to the spectrometer while flushing with pressurized N_2_ gas. The spectra were fitted with a Shirley background model [46], and had their photoelectron binding energies (BE) referenced to the C 1s adventitious carbon peak set at 285 eV BE. All fits used 70% Lorentzian and 30% Gaussian curves, specifically, the Fe 2p region was fitted with components for GS multiplets [47], surface structures and shake-up features [48]. Other XPS regions were subject to fitting with components previously reported in the literature; these fits are detailed further with the results.

Analysis of the Cr K edge by X-ray absorption spectroscopy (XAS) was performed on Beamline B18 at the Diamond Light Source (DLS). Spectra were obtained in transmission in a 10 minute top-up mode, with a ring current of 250 mA and an energy of 3 GeV. The Si(111) double crystal monochromator used was calibrated using Fe and Cr foil K edges, with first inflection points of 7112 eV and 5989 eV, respectively. Harmonic rejection of the monochromated radiation was achieved using two Pt coated mirrors at an incidence angle of 7 mrad. Prior to analyses, the samples were dried and manipulated under an N_2_ atmosphere and cooled using an LN2 cryostat (Oxford Instruments, Optistat DN2), with a PT100 sensor integrated in to the sample holder during data collection.

The X-ray absorption near-edge structure (XANES) and extended X-ray absorption fine structure (EXAFS) data were analyzed using ATHENA software (ver 0.9.13) [49,50]. The *E_o_* was obtained from the maximum of the first inflection and calibrated using the K edge of a Cr foil, collected simultaneously, with maximum of the first derivative set to 5989 eV. The spectra were then background subtracted, aligned, normalized and merged for each sample. The EXAFS spectra were then analyzed using ARTEMIS software for the IFEFFIT program [49]. The *k^3^* weighted Fourier Transform, applying a Hanning window, was fitted using theoretical parameters obtained from FEFF, from an inorganic crystal structure database, and applied to obtain statistically reasonable fits of the data.

### 2.6 Evaluation of reaction kinetics

The removal of aqueous Cr(VI) was described by a pseudo-1st order kinetic model, where the observed rate is proportional to the aqueous Cr(VI) concentration:

(1) $$\frac{\text{d}[\text{Cr}(\text{V I})]}{dt}=-k_{obs}[Cr(VI)]$$

where [Cr(VI)] is the concentration of aqueous Cr(VI), *t* is time and *k*_obs_ is the observed pseudo-1st order rate constant. These were calculated by the linear regression of ln[Cr(VI)] vs time (mins) and provide comparative Cr(VI) removal rates between the experiments with corresponding starting Cr(VI) concentrations. They are calculated from the Cr(VI) removal data over the entire course of the experiment, where active Cr(VI) removal occurs.

## 3 Results

### 3.1 Cr(VI) removal from model solutions – impact of pH and formate concentration

**Fig. 1:**
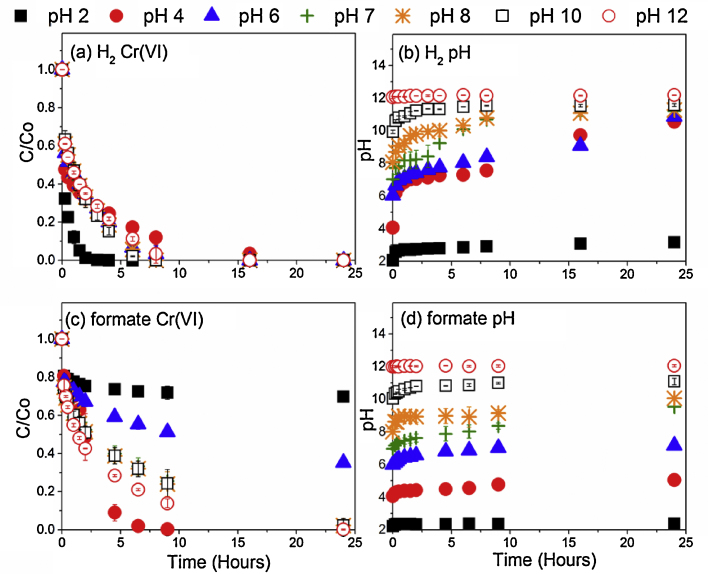
C/Co of aqueous Cr(VI) concentration over time, with H_2_ gas (a) and 100 mM formate (c) as respective electron donors, alongside pH over time for H_2_ gas (b) and 100 mM formate (d), upon amendment with 0.32 g L^−1^ Pd-BnM. Error bars represent standard deviation of duplicate experimental time series.

To better characterize Cr(VI) removal from model solutions using Pd-BnM, supplied with formate or H_2_, batch experiments were performed employing variable starting pH values (Fig. 1). Amendment of the starting pH of the reaction was found to exert less control over Cr(VI) removal behavior in the H_2_ experiment (Fig 1a) than those using formate as an electron donor (Fig 1c), where in the former, removal was consistent across most pH values, with optimum conditions recorded at pH 2. Cr(VI) removal was far more variable below pH 7, when formate was used, although performance was relatively consistent at near neutral to alkaline values. Increases in solution pH were noted during all treatments with both electron donors, with the exception of those with a starting pH of 12 (Fig. 1b and d). The presence of formate however limited increases in the pH of the solution, compared to the H_2_ amended experiment.

Experiments conducted using increasing starting concentrations of formate (0–200 mM), at pH 12, showed that a large excess of formate (>50 mM formate), in respect to the Cr(VI) concentration (0.5 mM) treated, was required for appreciable reaction kinetics (see S.I. Fig. S1). This experiment also showed that minimal quantities of formate were consumed over the reaction period.

### 3.2 Kinetics of Cr(VI) removal – Model pH 12 solutions and COPR leachate

**Fig. 2:**
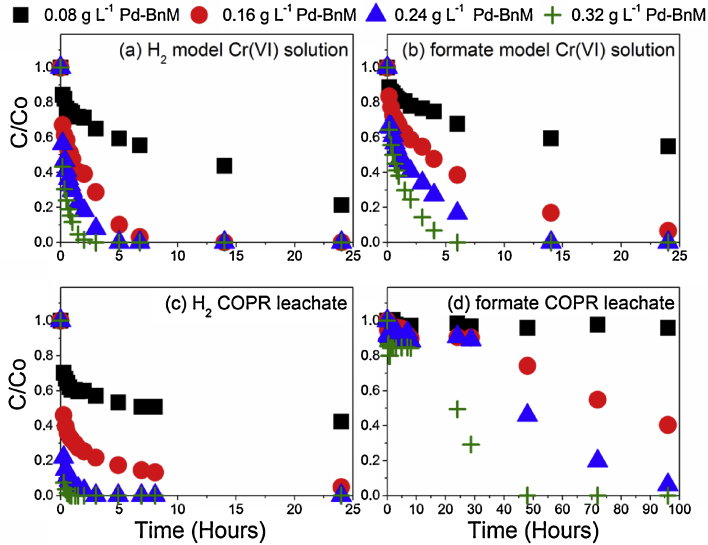
C/Co of aqueous Cr(VI) concentration over time with Pd-BnM/H_2_ gas in a model 0.5 mM Cr(VI) solution (a) and 0.5 mM Cr(VI) COPR leachate (c), and Pd-BnM/100 mM formate in a model Cr(VI) solution (b) and COPR leachate (d). Note the different time scale used for (d).

**Fig. 3:**
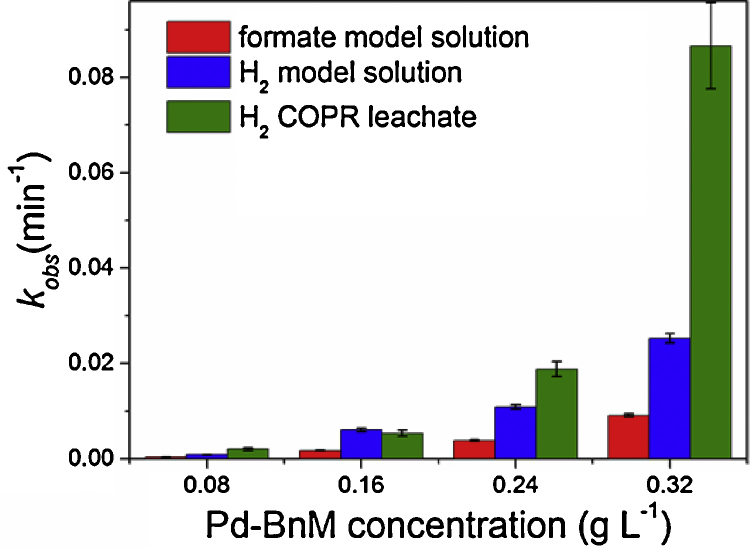
Pseudo-1st order reaction rate constants, *k*_obs_ (mins^−1^), of aqueous Cr(VI) removal with varying electron donor and reaction solution. Note poor Cr(VI) removal from COPR leachates during reaction using Pd-BnM/formate precluded the calculation of pseudo-1st order reaction rates. Error bars indicate the standard error of *k*_obs_ values, calculated from linear regression of data from Fig. 2 (see Fig. S2 for linear regressions and Table S2 for data).

The effect of Pd-BnM loadings on Cr(VI) removal from model pH 12 solutions and COPR leachate, using H_2_ and formate, are presented in Fig. 2 and their linear regression fits presented in Fig. S2 and Table S2. In model solutions, the Pd-BnM loading had a major control over the Cr(VI) removal (Fig. 2a and b) in both electron donor experiments; with increasing Pd-BnM loadings giving increasing Cr(VI) removal *k*_obs_ values (Fig. 3), in a non-linear fashion. At lower Pd-BnM loadings using formate, there was a marked slowing of the rate of reaction with time, resulting in poorer linear regression values (*r*^2^ = 0.90) of ln[Cr(VI)] vs *t* (S.I. Fig. S2 and Table S2).

When applied to the COPR leachate, the Pd-BnM/formate combination resulted in little removal of Cr(VI) over the first 24 h of reaction, followed by a slow rate of removal, dependent on Pd-BnM loadings, over the full 98 h of reaction time (Fig. 2d). These data did not adhere to the pseudo-1st order reaction model applied to the other experiments, precluding it from detailed comparisons of *k*_obs_ values. At most Pd-BnM loadings, considerably higher *k*_obs_ were obtained for Cr(VI) removal from the COPR leachate with H_2_ experiment, when compared to the equivalent treatment of model solutions (Fig. 2c and Fig. 3). Again the increase in *k*_obs_ with increasing Pd-BnM loading was found not to conform to a linear relationship.

Soluble COPR leachate components were quantified in supernatants before amendment with an electron donor, after electron donor amendment and after reaction with Pd-BnM (S.I. Table S3). The Si concentrations did not change appreciably, while the Ca levels marginally decreased after the addition of formate (9% loss). A greater decrease was observed at the end of the reaction with Pd-BnM, in all replicates, with a maximum loss in the formate supplemented experiments, by on average 32% and 15% for formate and H_2_, respectively. Concurrent to this, CO_3_^2−^ concentrations decrease to below detection limits when formate was used as the electron donor, while H_2_ gas amendment had minimal impact.

### 3.3 Maximum Cr(VI) removal – model pH 12 solutions and COPR leachate

Inactivation of the catalyst was noted for both electron donors, from model Cr(VI) solutions and COPR leachate, after reaction with excess Cr(VI) and electron donor. The maximum Cr(VI) removal levels, upon cessation of the reaction, in all experimental conditions are presented in Table 1. The highest level of Cr(VI) removal occurred in the model pH 12 solutions, with higher removal levels using formate compared to H_2_. Cr(VI) removal by Pd-BnM was less efficient when applied to the more chemically complex COPR leachate; removing 4% Cr(VI) and 64% Cr(VI) as from model solutions, for the formate and H_2_, respectively. The Pd-BnM/formate combination, as previously discussed for kinetic experiments, exhibited a dramatic loss in reactivity when applied to the COPR leachate compared to model Cr(VI) solutions.

### 3.4 Solid phase analysis – TEM and XPS

**Fig. 4:**
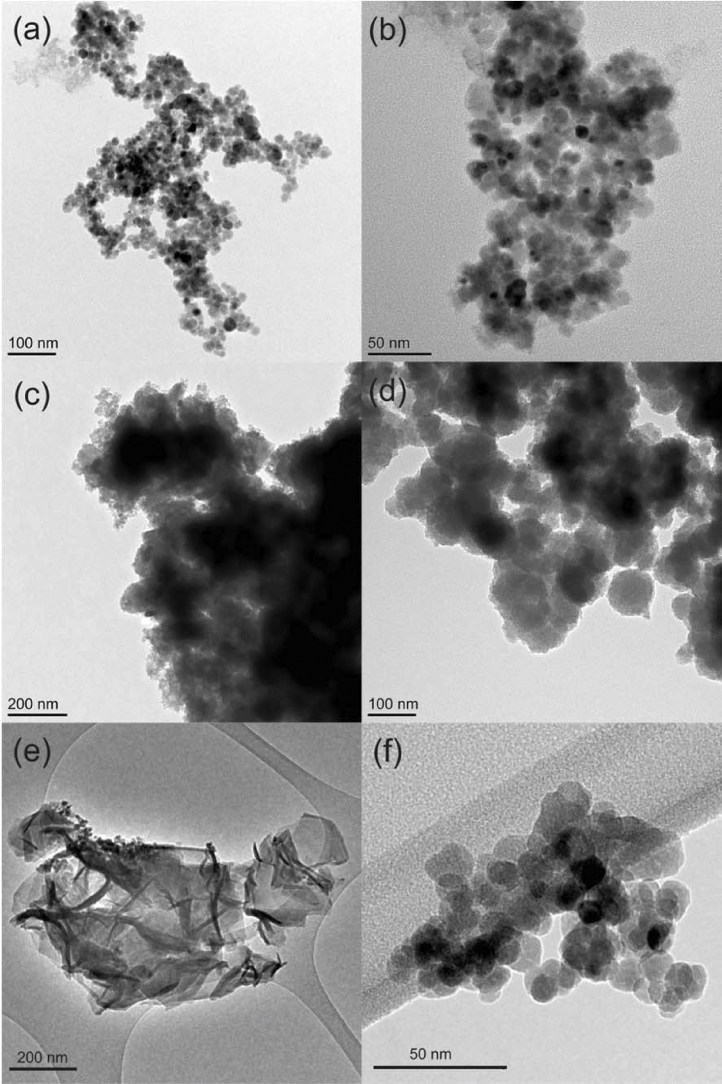
Transmission electron micrographs of Pd-BnM prior to reaction (a) and (b), upon terminal reaction in presence of excess H_2_ gas with a model Cr(VI) solution (c) and the COPR leachate (e), and in the presence of excess formate with a model Cr(VI) solution (d) and the COPR leachate (f). Note the differing scale bars in the images.

**Fig. 5:**
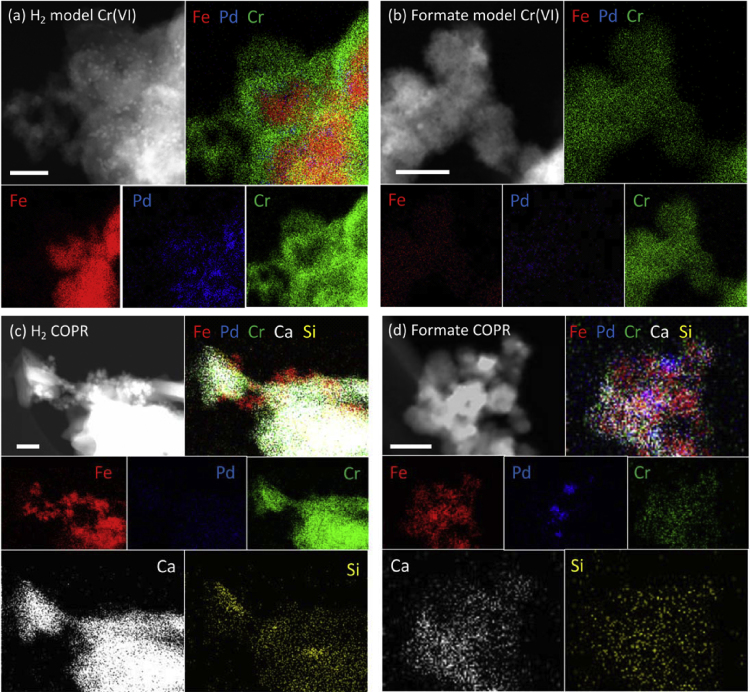
TEM-EDX elemental abundance maps of Cr, Fe, Pd, Ca and Si and their corresponding HAADF images (top left in each frame) for Pd-BnM after terminal reaction with a model Cr(VI) solution, in the absence of co-solutes, while in the presence of excess H_2_ gas (a) and formate (b), and the COPR leachate in the presence of excess H_2_ gas (c) and formate (d). Note the white bar for scale is 50 nm in length.

XPS (Table 1. and S.I. Fig. S3 and S4) and TEM (Figs. 4 and 5) analysis was used to probe the surface chemistry of the un-reacted and Cr(VI)-reacted (and inactivated) Pd-BnM. A TEM-EDX map of the un-reacted Pd-BnM is also presented in S.I. Fig. S5, exhibiting Pd rich clusters upon larger Fe rich particles. It should be noted that XPS analysis is a surface sensitive technique, targeting the upper <10 nm [51], and the results presented do not therefore reflect the bulk chemistry of the samples. The un-reacted Pd-BnM surface was composed of purely Fe and Pd (C and O subtracted) (Table 1), and in TEM images the individual magnetite nanoparticles (∼5–30 nm) are visible (Fig. 4a and b). Upon reaction with model solutions, using H_2_ or formate, larger micron-scale aggregates formed (Fig. 4c and d), which from TEM-EDX analysis are composed of a high abundance of Cr associated with the nano-scale Fe-Pd rich particles (Fig. 5a and b). Upon XPS analysis an Na component was also detected, likely from the addition of NaOH used to increase the pH of the solution (Table 1). Pd was not detected on the surface of any of the reacted samples by XPS analysis (Table 1), although it is visible in TEM-EDX maps (Fig. 5), and may be out of the sampling depth of XPS. COPR inactivated Pd-BnM samples, amended with H_2_, formed large sheet-like aggregates (Fig 4e), with a high abundance of Si and Ca associated with dense Fe particles (Fig. 5c and Table 1). XPS analysis of this sample observed decreased Fe and Cr proportions compared to data from samples reacted with the model solution (Table 1). The COPR reacted Pd-BnM/formate samples exhibited minimal morphological changes compared to the un-reacted particles (Fig 4f), with individual Pd-BnM nanoparticles visible. This sample also recorded lower abundances of Cr by XPS analysis, while maintaining significant Fe contributions alongside additional Ca and Si (Fig. 5d).

The post-treatment Cr 2p spectra (SI Fig. S3(d) and data in Table 1) were dominated by a peak with a Cr 2p_3/2_ peak BE of ∼577.5 eV, consistent with previously reported Cr(III) spectra [52]. While the COPR reacted samples were best fitted with this sole Cr(III) component, the samples that were reacted with model solutions also appeared to have a minor contribution from a Cr(VI) component; fitted at ∼579 eV. The Fe 2p spectra with multiplet fitting, and their corresponding % Fe(II), are presented in Table 1 and S.I. Fig. S3(a). The proportion of Fe(II) associated with the un-reacted Pd-BnM was marginally below that of stoichiometric magnetite (33%), at 29%. Upon reaction with Cr(VI) solutions, only the COPR Pd-BnM/formate sample contained high enough % Fe to allow fitting, recording a decrease in Fe(II) from 29% to 22%. The O 1 s region (SI Fig. S3(b)) was also fitted to O^2−^, —OH and H_2_O components at ∼530.1, ∼531.6 and ∼533.2 eV BE, respectively [53,54]. The un-reacted Pd-BnM was found to contain a distinct O^2−^ alongside–OH components, while upon reaction with model solutions the spectra were dominated by the —OH component. The COPR reacted samples were also dominated by the–OH component with contribution from a broad O^2−^ component. The Pd 3 d spectra of the un-reacted Pd-BnM (SI Fig. S3(c)) was successfully fitted to 2 components; of Pd(0) metal 335.1–335.9 eV BE and Pd(II) oxide 336.3–337.9 eV BE [55], primarily of the Pd(0) component. No fitting was possible to the XPS spectra from the solution inactivated samples, due to the low Pd contributions.

### 3.5 Solid phase analysis – synchrotron analyses

**Fig. 6:**
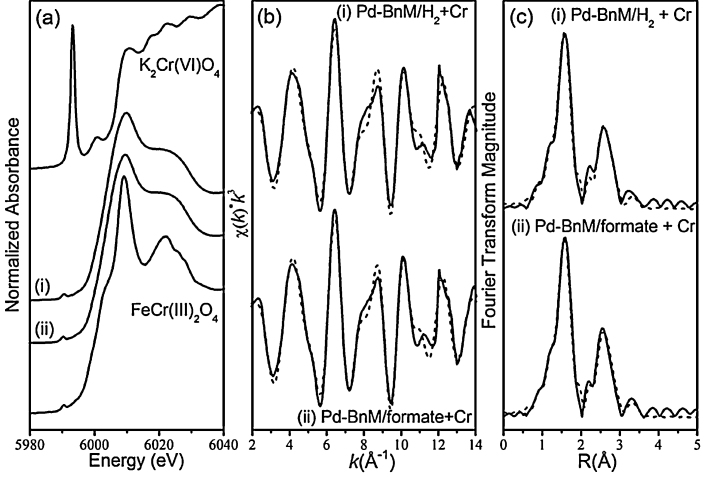
Cr K edge normalized XANES spectra (a), *k^3^* weighted EXAFS spectra (b) and corresponding Fourier transforms (c), for the Pd-BnM reacted with model Cr(VI) solutions and supplied with H_2_ gas (i) and formate (ii) as the electron donor. In all diagrams solid lines represent data and dashed lines represent model fits.

The Cr K edge XANES and EXAFS spectra with corresponding model fits of the Pd-BnM, after reaction with model Cr(VI) solutions, and in the presence of H_2_ or formate, are presented in Fig. 6 and Table 2. The XANES spectra of the reacted Pd-BnM samples lack the prominent pre-edge feature of the Cr(VI) standard (K_2_CrO_4_) at ∼5996 eV [56], but do contain the pre-edge feature at ∼5992 eV associated with Cr(III) [57].

The EXAFS spectra were best fitted with a first shell of 6 O atoms, with a single Cr-O interatomic distance of 1.97 and 1.98 Å, for the H_2_ and formate samples, respectively (Table 2). A second shell was fitted in co-ordination with 4 Cr-Cr/Fe single scatterers at 3.01 Å, where EXAFS is unable to resolve between Cr and Fe as the difference in atomic numbers is ≤2 [58]. The fit for both samples was improved further by the addition of a 3rd shell of 2 backscattering Cr-Cr/Fe atoms at 3.61 and 3.60 Å, for H_2_ and formate samples, respectively.

## 4 Discussion

### 4.1 Removal of aqueous Cr(VI) – model solutions

Heterogeneous catalysts are widely reported to react with substrates via an initial adsorption of the electron donor to an active Pd(0) site, followed by heterolytic or homolytic fission, charging the Pd(0) with reactive H•[2,59]. In the case of Cr(VI) reduction, this is then followed by the co-adsorption of the Cr(VI) anion and reaction with H•. As the speciation of the Pd-BnM surface, Cr(VI) oxyanions and the formate are pH dependent, a complex relationship is likely to exist between their electrostatic interactions over the pH range. Under acidic conditions, where the H_2_ electron donor is most efficient (pH 2) and the formate system highly variable, with both most inhibited (pH 2) and optimal removal observed (pH 4), the surface of the Pd-BnM is likely to be more electro-positively charged, while the dominant Cr(VI) anion is likely to be the negative HCrO_4_^−^ [60]. The increased attraction of the positive surface and negative Cr(VI) anion potentially accounts for the increased reaction rate in the H_2_ experiment under acidic conditions. The complex behavior when employing formate is potentially due to the speciation of the formate, which is in equilibrium with formic acid (p*K_A_* = 3.75) [5], with inhibition coinciding with the increased dominance of formic acid. At near neutral and moving to the environmentally relevant alkaline pH conditions, deprotonated CrO_4_^2−^ and HCOO^−^ will dominate, while the Pd-BnM surface is likely to be increasingly electro-negatively charged; where previous studies using synthetic Pd(0) on magnetite recorded a point of zero charge (pzc) of 7 [61]. Despite these conditions which are expected to favor the repulsion of similarly negatively charged Pd-BnM surface and reactant anions, in the high pH range tested, no obvious increasing loss in reactivity was observed. This is potentially a result of a concurrent increase in dispersion, due to electrostatic repulsion between Pd-BnM particles, which will help to maximize the reactive surface of the particles. This is all the more significant due to the observed generation of alkalinity which occurs by the liberation of OH^−^ during the reduction of the Cr(VI) anions [39], where, however, the formate does appear to act as a buffer limiting pH change.

The *k*_obs_ kinetic data for Cr(VI) reduction and removal from model Cr(VI) solution, with increasing Pd-BnM loadings, indicates that catalyst concentration exerts a strong control. As the reaction is mediated by Pd(0) content, and its availability as a reactive surface, increasing Pd-BnM loadings would enable more efficient coupling of H• to Cr(VI), increasing reaction rates. The non-linear relationship observed between Pd-BnM loading and *k*_obs_, for both electron donors, most likely represents a reactive surface limited system in the lowest Pd-BnM loading experiments. It is also highly likely that the reductive precipitation of Cr, implicated in investigations in to catalyst inactivation, limits *k*_obs_ values by further decreasing reactive surface area.

Magnetite, used as the support for the catalyst in this study, has previously been investigated in regards to its reactivity towards Cr(VI), via its surface Fe(II) content [17,57,62]. However the maximum levels of Cr(VI) removal from model solutions by the Pd-BnM presented here, using both formate or H_2_, are far greater than stoichiometrically possible using un-functionalized magnetite, ∼75 mg Cr(VI) g^−1^ magnetite assuming complete consumption of the electrons available by Fe(II). The biogenic magnetite, employed as the carrier particle in this study, has previously been used to treat pH 12 Cr(VI) solutions, and recorded a removal of 32 mg Cr(VI) g^−1^ magnetite due to passivation of the particles reactive surface [43]. In addition, other studies have also found the functionalization by Pd(0) greatly increased the potential Cr(VI) removal compared to magnetite [17]. It should also be noted that a no electron donor control was performed and is presented in S.I. Fig S1, this showed minimal removal of Cr(VI) by Pd-BnM, at the catalyst concentration used, when compared to experiments conducted in the presence of the electron donor formate.

Inactivation of catalysts by a variety of co-solutes has been demonstrated previously for the treatment of halogenated solvents [61], although inactivation by Cr has received little attention. The inactivation of the Pd-BnM in model solutions is possibly due to the accumulation of the reduced Cr(III) phase upon the surface, noted in TEM-EDX and XPS analysis. This phase formed over the surface of the Pd-BnM and is likely to act as an insulating layer, limiting surface mediated contact between the Cr(VI) or electron donor and the reactive Pd-BnM surface. This process is likely to be similar to the passivation reported for magnetite and zero valent iron (ZVI) treatment of Cr(VI) [58,63,64].

Upon analysis of the reacted Cr(III) phase on the Pd-BnM, the Cr K edge XANES spectra bear more resemblance to those previously reported for Cr(OH)_3_ and CrOOH [65,66], lacking the edge feature of the spinel FeCr_2_O_4_ standard presented here. Further to this, EXAFS analyses and subsequent fitting of data from the samples of Pd-BnM formate and H_2_ reacted with model Cr(VI) solutions, indicated that the same Cr phase forms irrespective of the electron donor used. The fitted Cr-O shell (1.97–1.98 Å) is consistent with a Cr(III) octahedral co-ordination, where Cr(VI) typically forms a tetrahedral co-ordination at shorter interatomic distances of 1.67–1.69 Å [54,58,67]. Considering the TEM-EDX maps which indicate overgrowth of the Fe surface with a discreet Cr phase, the two outer shells (3.01 and 3.60–3.61 Å) are likely to be Cr-Cr. The first Cr-Cr/Fe shell (3.01 Å) is consistent with the edge sharing distances reported for polymeric CrOOH polymorphs at 3.00–3.06 Å [57,68–70]. Significantly the fitted spectra lack the corner sharing Cr-Cr shell, at ∼3.98 Å [54,68], common to ɣ-CrOOH. The second fitted Cr-Cr/Fe shell (3.60–3.61 Å) has been interpreted previously as a double corner sharing path between adsorbed Cr(III) and Fe(III) hydroxides [70,71]. It is also pertinent that the fitted spectra lack the larger Cr-Cr atomic distance shells associated with chromite [58]. This supports TEM observations and previous studies [17], which suggest that the majority of Cr in such systems is in a non-magnetic surface phase, as opposed to incorporated into a spinel structure [72].

### 4.2 Removal of Cr(VI) – COPR leachate

The chemical composition of the COPR leachate employed here reflects the cementitous nature of the COPR [73]; with a highly alkaline pH and containing aqueous Cr, Ca, Si and CO_3_^2−^ [31,34,74]. The presence of co-solutes has been implicated previously in inactivation and inhibitory processes during treatment by Pd(0) catalysts [61,75–77].

By comparison of Cr removal data obtained from Pd-BnM/formate treating COPR and model solutions, a significant inhibition of removal with the COPR was observed. We suggest that this inhibition is related to the presence of the co-solutes Ca^2+^ and CO_3_^2−^. Upon addition of formate to the COPR system, the complete removal of CO_3_^2−^ and the partial removal of Ca indicate precipitation of carbonate species, e.g. CaCO_3_. Although assuming a 1:1 stoichiometric ratio of Ca^2+^ to CO_3_^2−^, it cannot fully account for the loss of CO_3_^2−^. This behavior is potentially responsible for the loss of catalytic activity in the COPR Pd-BnM/formate experiments by blocking catalyst interactions by surface precipitation. Such precipitation processes have been noted previously to lead to a decrease in reaction rates and a loss in Cr(VI) removal capacity in ZVI permeable barriers [78,79]. The impact of Ca^2+^ and CO_3_^2−^ was explored further in experiments using model Cr(VI) solutions, via the addition of the major leachate co-solutes detailed in S.I. Text S2. These experiments implicated the presence of CaCO_3_ in the inhibition of Cr(VI) removal, while Ca^2+^ or CO_3_^2−^ in isolation did not, S.I. Fig. S6, S7 and S8 and S.I. Table S4.

In contrast, we noted significant promotion, in comparison to model solutions, of *k*_obs_ values at higher Pd-BnM loadings in the H_2_ COPR experiment. Here changes in the solution chemistry suggest a limited role for precipitation of carbonate species. The co-solute experiment, again detailed in S.I. Text S2, implicated the Ca^2+^ cation in promotion of Cr(VI) removal rates, interestingly in the absence of CO_3_^2−^ the formate system also exhibited a promotion effect, S.I. Fig. S6–S8 and S.I. Table S4. The causes of this Ca^2+^-mediated promotion are however unclear. This promotion effect in the COPR experiment did not extend to the lower Pd-BnM loadings, where a slowing of the rate over the experiment was also noted. At lower Pd-BnM loadings this is interpreted as a reactive surface limitation effect, with increasing passivation of the surface with the Cr, Ca and Si, as seen in TEM-EDX maps and XPS data.

The maximum levels of Cr(VI) removal from COPR with the Pd-BnM/H_2_ treatment, represent far greater removals than previously reported for micron-scale and nano-scale zero valent iron (ZVI) from COPR groundwater, of 1 and 73 mg Cr(VI) g^−1^ Fe(0), respectively [80]. The removals are also far in excess of those previously reported for the unfunctionalized biogenic magnetite, which were able to remove 24 mg Cr(VI) g^−1^ magnetite from COPR groundwater [43]. The greater removal reported here (352 mg Cr(VI) g^−1^ Pd-BnM) is a result of the sustained catalytic reactivity in the presence of the electron donor H_2_, as opposed to the finite electron source Fe(0) of the ZVI or the Fe(II) of magnetite. However catalyst inactivation, after removing appreciable quantities of Cr(VI), was noted for all treatments in both model and COPR solutions. The increased complexity of the COPR solution is inferred to be responsible for the decrease in total Cr(VI) removals by the Pd-BnM. As previously discussed, when employing formate, this is evident as a loss of catalytic activity, potentially mediated by CaCO_3_ precipitation. The ∼50% decrease in maximum Cr(VI) removal, compared to the model solution, during the Pd-BnM/H_2_ experiment, is also likely to be a result of the more complex chemistry of the COPR. As seen from TEM-EDX maps and XPS data there is a significant presence of Ca and Si on the surfaces, likely to increase the passivation of the surface, again, as previously seen in both magnetite and ZVI systems [43,74]. It should be noted that these experiments, performed with limited Pd-BnM and an excess of both Cr(VI) and co-solutes, reflect a limited surface system where the co-solutes, with a contribution from Cr(VI), are able to passivate the surface. This experimental set up was chosen to imitate a sustained reaction scenario where Cr(VI) and co-solutes would be re-supplied until inactivation of the catalyst. It is however unclear if an increased Pd-BnM surface was employed, which is able to attenuate the non-target co-solutes, would still maintain a reactive Pd(0) surface leading to sustained Cr(VI) removal.

In conclusion, pH was found to have a major control over the efficiency of aqueous Cr(VI) removal when formate was used as an electron donor for Pd-BnM-mediated metal reduction, while the system was less sensitive to pH effects when H_2_ was used as the electron donor. At environmentally relevant alkaline pH conditions, challenged with model Cr(VI) solutions, electron donors coupled with Pd-BnM were able to remove aqueous Cr(VI) efficiently by reduction to Cr(III). In time, this led to catalyst inactivation, most likely due to the formation of an insulating surface CrOOH phase. In the more complex solution chemistry of the COPR leachates, significant inhibition was noted in the presence of formate, while Cr(VI) removal rates were enhanced in the H_2_ experiment. Cr(VI) removal in model solutions, with combinations of co-solutes implicated the formation of CaCO_3_ in inhibition in the formate experiment, while the presence of Ca^2+^ (in the absence of carbonate) resulted in promotion of the catalytic reaction. The higher solute loading in COPR leachates significantly decreased the maximum Cr(VI) removal possible by the Pd-BnM/H_2_ experiment, inferred to be the result of the co-solutes Ca and Si occupying significant proportions of the catalyst surface, while completely inhibiting catalytic activity in the formate experiment.

The data published in this study illustrate the clear potential of biotechnologically engineered Pd(0)-bearing nanocatalysts for the remediation of Cr(VI) from contaminated waters at environmentally relevant alkaline conditions. Although catalyst inactivation was noted, the quantities of Cr(VI) removal, prior to loss of reactivity, from the model alkaline solutions and COPR leachates (in the Pd-BnM/H_2_ experiment) are far greater than those reported under similar conditions using conventional nZVI treatments [80]. The findings of this study also highlight the importance of the electron donor used, with superior performance using H_2_, compared to the formate-driven experiments, where significant inhibition was noted in COPR leachates.

The reactive life time of the catalyst, and potential for re-activation, are of importance when considering the cost and effectiveness of catalysts for contaminant remediation. Although several catalyst water treatments have reached field scale application [81–84], all have targeted organic contaminants which, unlike the reductive precipitation of Cr(VI), do not generate products directly implicated in catalyst deactivation. These studies did however note a loss of efficiency of the catalyst upon long term deployment, with various reactivation treatments used to regenerate the catalyst. The relatively high expense of Pd, where it makes up ∼2% by mass of the Pd-BnM particles, means further investigations on reactivation of the inactivated catalyst, where its magnetic properties are likely to aid its retrieval, are warranted.

## Figures and Tables

**Table 1:** Summary of XPS data obtained from the surface (<10 nm) of un-reacted and Cr(VI) reacted Pd-BnM.

	Total Cr(VI) removed from solution mg per g^−1^ Pd-BnM	XPS elemental composition (atomic %)						XPS		
		Fe	Pd	Cr	Na	Si	Ca	Pd metal: Pd oxide	Fe(II): Fe(III)	Cr(III): Cr (VI)
										
Pd-BnM unreacted	–	95	5	0	0	0	0	70: 30	29: 71	–
Pd-BnM/formate + Cr(VI) model	753 ± 38	4	0	50	45	0	0	–	–	91: 9
Pd-BnM/H_2_ + Cr(VI) model	551 ± 19	5	0	57	39	0	0	–	–	90: 10
Pd-BnM/formate + COPR	30 ± 5	13	0	5	0	28	54	–	22: 78	100: 0
Pd-BnM/H_2_ + COPR	352 ± 32	0	0	7	0	40	53	–	–	100: 0

**Table 2:** Fitting parameters from least square fits to *k*^3^ weighted Cr K edge EXAFS spectra.^a^

Sample	Shell	R (Å)	*N*	*σ*^2^	*R*-factor
Pd-BnM/H_2_ + Cr pH12	Cr-O	1.97	6	0.0028	1.9
	Cr-Cr/Fe	3.01	4	0.0064	
	Cr-Cr/Fe	3.61	2	0.0096	
Pd-BnM/formate + Cr pH12	Cr-O	1.98	6	0.0029	1.7
	Cr-Cr/Fe	3.01	4	0.0064	
	Cr-Cr/Fe	3.60	2	0.0090	

a Absorber – backscatterer interatomic distance (*R*), number of backscattering atoms in relation to the Cr adsorber, Debye–Waller factor (*σ*^2^).
