# Supplementary material for: Effective treatment of alkaline Cr(VI) contaminated leachate using a novel Pd-bionanocatalyst: Impact of electron donor and aqueous geochemistry
Source: Appl Catal B. 2015 Jul;170-171:162–72. doi: 10.1016/j.apcatb.2015.01.017 (PMC4394151; doi:10.1016/j.apcatb.2015.01.017)
Supplement: Supplementary file 1 [file mmc1.docx]

Supporting Information for “Effective treatment of alkaline Cr(VI) contaminated leachate using a novel Pd-bionanocatalyst: impact of electron donor and aqueous geochemistry”

Mathew P Watts^1#^, Victoria S. Coker^1^, Stephen A. Parry^2^, Russell A. P. Thomas^3^, Robert Kalin^4^ & Jonathan R. Lloyd^1^*

1 School of Earth, Atmospheric and Environmental Sciences and Williamson Research Centre for Molecular Environmental Science, The University of Manchester, Manchester, M13 9PL, U.K. 2 Diamond Light Source, Chilton, Didcot, Oxfordshire, OX11 ODE, U.K. 3 Parsons Brinckerhoff, Queen Victoria House, Redland Hill, Bristol, U.K. 4 Department of Civil and Environmental Engineering, James Weir Building, University of Strathclyde, Glasgow G1 1XJ, UK.

^#^ Current address: School of Earth Sciences, University of Melbourne, Victoria 3010, Australia.

* Jon.Lloyd@.manchester.ac.uk

**S.I. Text 1 - Biogenic magnetite synthesis**

The initial Fe(III) phase of amorphous 2-line ferrihydrite was produced according to the method detailed in [1]. A late log phase culture of *G. sulfurreducens* was prepared under an atmosphere of N_2_-CO_2_ (80:20), using 20 mM acetate and 40 mM fumerate as electron donor and acceptor respectively [2]. The cells were washed three times and incubated, with an optical density (OD) 600 nm of 0.4, with 50 mM of 2-line ferrihydrite, 20 mM Na acetate, 30 mM NaHCO_3_ buffer and 10 μM 9,10-anthraquinone-2,6-disulfonate (AQDS) as an electron shuttle, at 30°C in the dark for 1 week. The resulting slurry was magnetically separated and washed using anoxic 18.2 MΩ water and stored at 4°C until use.

| Table S1. COPR leachate composition. | |
| --- | --- |
| **Component** | **Concentration mM (mg L^-1^)** |
| Ca | 15.36 (583) |
| Cr(total) | 0.48 (25) |
| Cr(VI) | 0.50 (26) |
| Si | 0.06 (2) |
| Carbonate | 12.94 (777) |
| pH | 11.98 |





Fig. S1. C/Co of aqueous Cr(VI) concentration (a) and formate concentration (b) vs. time at varying starting concentrations of formate, at pH 12. The starting Cr(VI) concentration was 0.5 mM and Pd-BnM addition was equivalent to 0.24 g L^-1^ in all replicates. Error bars indicate the standard deviation of duplicate experimental time series.





Fig. S2. ln[Cr(VI)] over time for the increasing Pd-BnM addition batch experiments reacted with a pH 12 model Cr(VI) solution using H_2_ and formate as the electron donor and with COPR leachate using H_2_ gas as the electron donor. The poor removal of Cr(VI) during COPR leachates treatment with Pd-BnM/formate precluded this data from reaction rate fitting.

| Table S2. Aqueous Cr(VI) removal rates as a function of Pd-BnM addition, selection of electron donor and reaction media. | | | | | | | | | | | |  |  |
| --- | --- | --- | --- | --- | --- | --- | --- | --- | --- | --- | --- | --- | --- |
| **Experimental Conditions** | | **Pd-BnM** | **Formate** | | | ***k_obs_* (mins^-1^)** | | **Standard error *k_obs_* (mins^-1^)** | **r^2^** | | **Data points** |  |  |
| Formate - Variable Pd-BnM addition (model solution) | | 0.08 g/L | 100 mM | | | 0.0003 | | 0.00003 | 0.90 | | 13 |  |  |
|  |  | 0.16 g/L | 100 mM | | | 0.0017 | | 0.00004 | 0.99 | | 13 |  |  |
|  |  | 0.24 g/L | 100 mM | | | 0.0038 | | 0.00016 | 0.98 | | 11 |  |  |
|  |  | 0.32 g/L | 100 mM | | | 0.0091 | | 0.00028 | 0.99 | | 10 |  |  |
| H_2_ gas - Variable Pd-BnM addition (model solution) | | 0.08 g/L | - | | | 0.0009 | | 0.00004 | 0.98 | | 13 |  |  |
|  |  | 0.16 g/L | - | | | 0.0061 | | 0.00037 | 0.97 | | 10 |  |  |
|  |  | 0.24 g/L | - | | | 0.0109 | | 0.00043 | 0.99 | | 9 |  |  |
|  |  | 0.32 g/L | - | | | 0.0253 | | 0.00099 | 0.99 | | 6 |  |  |
| H_2_ gas - Variable Pd-BnM addition (COPR extract) | | 0.08 g/L | - | | | 0.0020 | | 0.00030 | 0.79 | | 13 |  |  |
|  |  | 0.16 g/L | - | | | 0.0053 | | 0.00062 | 0.91 | | 8 |  |  |
|  |  | 0.24 g/L | - | | | 0.0188 | | 0.00154 | 0.96 | | 8 |  |  |
|  |  | 0.32 g/L | - | | | 0.0866 | | 0.00902 | 0.98 | | 3 |  |  |
|  | |  |  | | |  | |  |  | |  |  |  |
| Table S3. Concentration of groundwater components. | | | | | | | | | | | | | |
| **Component** | | **Pre addition -Conc mM** | | | **Electron Donor** | **Pd-BnM addition (g L^-1^)** | | **Post electron donor addition - Concentration mM** | | | **Final timepoint - Concentration mM** | | |
| Ca | | 15 | | | H_2_ | 0.08 | | 15 | | | 12 | | |
|  |  |  |  |  | H_2_ | 0.16 | | 15 | | | 13 | | |
|  |  |  |  |  | H_2_ | 0.24 | | 15 | | | 13 | | |
|  |  |  |  |  | H_2_ | 0.32 | | 15 | | | 13 | | |
|  |  |  |  |  | formate | 0.08 | | 14 | | | 11 | | |
|  |  |  |  |  | formate | 0.16 | | 15 | | | 10 | | |
|  |  |  |  |  | formate | 0.24 | | 12 | | | 11 | | |
|  |  |  |  |  | formate | 0.32 | | 13 | | | 9 | | |
|  | |  | | |  |  | |  | | |  | | |
| Si | | 0.06 | | | H_2_ | 0.08 | | 0.06 | | | 0.06 | | |
|  |  |  |  |  | H_2_ | 0.16 | | 0.06 | | | 0.06 | | |
|  |  |  |  |  | H_2_ | 0.24 | | 0.06 | | | 0.06 | | |
|  |  |  |  |  | H_2_ | 0.32 | | 0.06 | | | 0.06 | | |
|  |  |  |  |  | formate | 0.08 | | 0.06 | | | 0.07 | | |
|  |  |  |  |  | formate | 0.16 | | 0.06 | | | 0.07 | | |
|  |  |  |  |  | formate | 0.24 | | 0.06 | | | 0.06 | | |
|  |  |  |  |  | formate | 0.32 | | 0.06 | | | 0.06 | | |
|  | |  | | |  |  | |  | | |  | | |
| CO_3_^2-^ | | 13 | | | H_2_ | 0.08 | | 14 | | | 16 | | |
|  |  |  |  |  | H_2_ | 0.16 | | 12 | | | 16 | | |
|  |  |  |  |  | H_2_ | 0.24 | | 13 | | | 16 | | |
|  |  |  |  |  | H_2_ | 0.32 | | 14 | | | 16 | | |
|  |  |  |  |  | formate | 0.08 | | n.a. | | | n.a. | | |
|  |  |  |  |  | formate | 0.16 | | n.a. | | | n.a. | | |
|  |  |  |  |  | formate | 0.24 | | n.a. | | | n.a. | | |
|  |  |  |  |  | formate | 0.32 | | n.a. | | | n.a. | | |
|  | |  | | |  |  | |  | | |  | | |

**
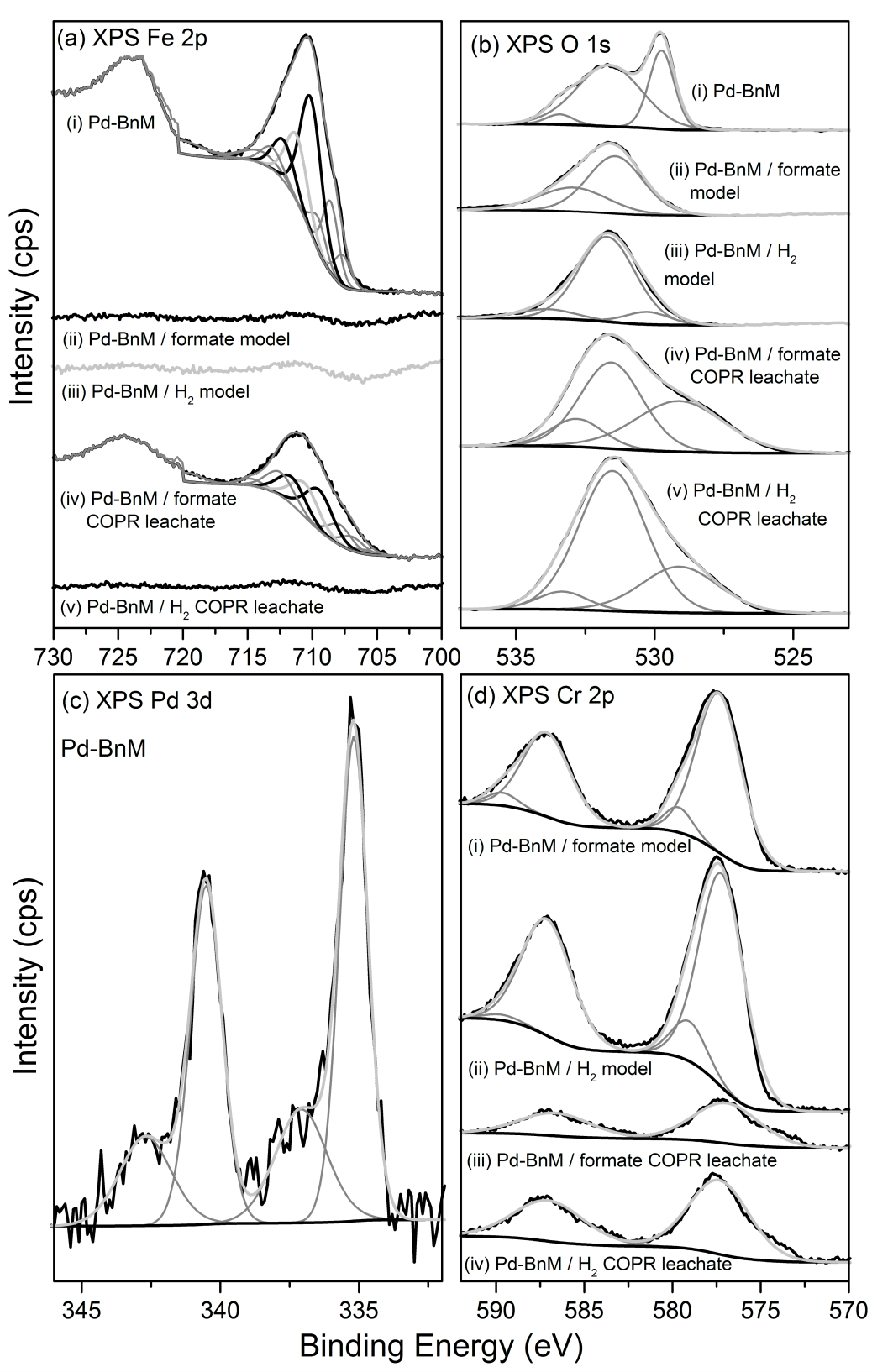
**

Fig. S3. X-ray photoelectron spectra of the Fe 2p (a), O 1s (b), Pd 3d (c) and Cr 2p (d) regions of the un-reacted and the model and COPR leachate reacted Pd-BnM using either H_2_ or formate as the electron donor.





Fig. S4. X-ray photoelectron spectra wide scans of the un-reacted and the model and COPR leachate reacted Pd-BnM using either H_2_ or formate as the electron donor. Note the appearance of Cr and Na upon reaction with the model solutions and additional Ca and Si upon reaction with COPR leachate.

**
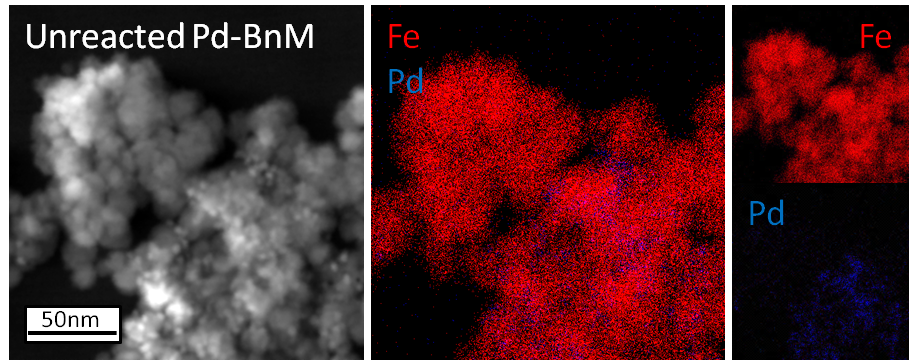
**

Fig. S5. STEM-EDX spot elemental abundance maps of Fe and Pd (centre and right), and their corresponding HAADF image (left) for the un-reacted Pd-BnM.

**S.I. Text 2 - Effect of co-solutes on Cr(VI) removal rates.**

The co-solute experiment again used a model solution of 0.5 mM Cr(VI) at pH 12, with additions of identified co-solutes in the COPR. Where possible these were added at the same concentrations as those identified in the COPR leachate. However, some concentrations were reduced due to poor solubility; 5 mM NaCO_3_, 0.5 mM CaCO_3_, 15 mM Ca(OH)_2_ or 0.1 mM SiO_2_. These serum bottles were subject to an addition of Pd-BnM stock to create a final concentration of 0.32 g L^-1^.

The impact of co-solutes on the Cr(VI) removal rate constants (*k_obs_*) from model pH 12 solutions are presented in S.I. Figure S6, S7 and S8. In comparison to when Cr(VI) in isolation (without co-solutes), Si and CO_3_^2-^ additions appeared to result in a minor promotion of rates for during both either electron donor experiments. The presence of CaCO_3_ had little impact in the H_2_ experiment but had an inhibitory effect upon the formate experiment, with slowing of the rate noted over the time course, and resulting in a lower r^2^ value (S.I. Table S4). The addition of Ca had the largest impact upon removal rates with significant promotion in the H_2_ experiment, and to a lesser extent the formate.





Fig. S6. C/Co of aqueous Cr(VI) concentration over time with H_2_ gas (a) and 100 mM formate (b), in the presence of a variety of co-solutes added to a model Cr(VI) solution at pH 12. The concentrations of the additional solutes are discussed in the S.I. Text 2.





Fig. S7. ln[Cr(VI)] over time for the removal of Cr in the presence of co-solutes from batch experiments reacted at pH 12 with model Cr(VI) solution using H_2_ and formate as the electron donor.





Fig. S8. Pseudo-1^st^ order reaction rate constants (mins^-1^) of aqueous Cr(VI) removal with varying electron donors and co-solutes. Note the change in scale above the break for the H_2_ data due to the comparatively high *K_obs_* recorded in presence of Ca. Error bars indicate the standard error of *K_obs_* calculation.

| Table S4. Calculated aqueous Cr(VI) removal rate constants in the presence of co-solutes. | | | | | |
| --- | --- | --- | --- | --- | --- |
| **Hydrogen Donor** | **Constituent** | ***K_obs_* (mins^-1^)** | **Standard error *K_obs_*** | **r^2^** | **Data points** |
| H_2_ | Cr only | 0.010 | 0.0006 | 0.98 | 8 |
|  | CO_3_ | 0.017 | 0.0011 | 0.97 | 8 |
|  | Ca | 0.212 | 0.0174 | 0.98 | 4 |
|  | Si | 0.010 | 0.0008 | 0.96 | 7 |
|  | CaCO_3_ | 0.011 | 0.0009 | 0.96 | 7 |
| Formate | Cr only | 0.004 | 0.0002 | 0.98 | 11 |
|  | CO_3_ | 0.005 | 0.0003 | 0.97 | 9 |
|  | Ca | 0.012 | 0.0003 | 0.99 | 9 |
|  | Si | 0.005 | 0.0002 | 0.99 | 10 |
|  | CaCO_3_ | 0.002 | 0.0002 | 0.94 | 9 |

**References**

[1] D.R. Lovley, E.J.P. Phillips, Availability of ferric iron for microbial reduction in bottom sediments of the freshwater tidal potomac river, Appl. Environ. Microbiol., 52 (1986) 751-757.

[2] D.R. Lovley, E.J.P. Phillips, Novel mode of microbial energy metabolism: Organic carbon oxidation coupled to dissimilatory reduction of iron or manganese, Applied and Environmental Microbiology, 54 (1988) 1472-1480.
